# Autophagy is essential for optimal translocation of iron to seeds in Arabidopsis

**DOI:** 10.1093/jxb/ery388

**Published:** 2018-11-04

**Authors:** Mathieu Pottier, Jean Dumont, Céline Masclaux-Daubresse, Sébastien Thomine

**Affiliations:** 1 Institut de Biologie Intégrative de la Cellule (I2BC), CEA, CNRS, Université Paris-Sud, Université Paris-Saclay, Avenue de la Terrasse, 91198 Gif-sur-Yvette, France; 2 UT2A, Hélioparc Pau Pyrénées, 2, avenue du président Angot, 64053 Pau, France; 3 Institut Jean-Pierre Bourgin, INRA, AgroParisTech, CNRS, Université Paris-Saclay, 78000, Versailles, France

**Keywords:** ^57^Fe, iron loading, iron recycling, leaf senescence, metal, remobilization, premature senescence

## Abstract

Micronutrient deficiencies affect a large part of the world’s population. These deficiencies are mostly due to the consumption of grains with insufficient content of iron (Fe) or zinc (Zn). Both *de novo* uptake by roots and recycling from leaves may provide seeds with nutrients. Autophagy, which is a conserved mechanism for nutrient recycling in eukaryotes, was shown to be involved in nitrogen remobilization to seeds. Here, we have investigated the role of this mechanism in micronutrient translocation to seeds. We found that *Arabidopsis thaliana* plants impaired in autophagy display defects in nutrient remobilization to seeds. In the *atg5-1* mutant, which is completely defective in autophagy, the efficiency of Fe translocation from vegetative organs to seeds was severely decreased even when Fe was provided during seed formation. Combining *atg5-1* with the *sid2* mutation that counteracts premature senescence associated with autophagy deficiency and using ^57^Fe pulse labeling, we propose a two-step mechanism in which Fe taken up *de novo* during seed formation is first accumulated in vegetative organs and subsequently remobilized to seeds. Finally, we show that translocation of Zn and manganese (Mn) to seeds is also dependent on autophagy. Fine-tuning autophagy during seed formation opens up new possibilities to improve micronutrient remobilization to seeds.

## Teaser

Autophagy is an essential recycling mechanism making micronutrients available in vegetative organs for subsequent reallocation to seeds

## Introduction

Metal micronutrients are essential to all forms of life. For instance, iron (Fe) plays a major role in oxido-reduction reactions allowing respiration in mitochondria and photosynthesis in chloroplasts (Nouet *et al.*, 2011). Worldwide, ~2 billion people suffer from Fe deficiency which affects mostly children and women in developing countries (WHO, 2016). Staple food crops are poor sources of Fe, and the major place of these crops in the human diet is one of the leading causes of Fe deficiency (Murgia *et al.*, 2012). During the last decade, different biofortification strategies such as fertilization, conventional breeding, and genetic engineering were pursued in an attempt to increase key micronutrient levels in seeds of crop species (Murgia *et al.*, 2012).

The improvement of micronutrient loading in seeds requires knowledge of micronutrient trafficking pathways within the plant, including uptake from soil and remobilization from senescing organs. To date, most studies on micronutrients have focused on their uptake in root cells, intracellular partitioning, and long-distance transport. Several proteins participate in the mandatory solubilization and reduction of Fe^3+^ to Fe^2+^ prior to its uptake by a plasma membrane transporter, AtIRT1, in the epidermis of Arabidopsis roots (Robinson *et al.*, 1999; Vert *et al.*, 2002; Santi and Schmidt, 2009; Fourcroy *et al.*, 2014; Lefèvre *et al.*, 2018). Fe is transported to the shoot upon loading in the xylem (Durrett *et al.*, 2007; Morrissey *et al.*, 2009), and translocation to sink tissues is mediated by loading/unloading in the phloem after Fe chelation to nicotianamine (Schuler *et al.*, 2012). Finally, the oligopeptide transporter 3 (AtOPT3) could transport chelated Fe to ensure Fe loading to seeds (Stacey *et al.*, 2008). During vegetative growth, Fe is mostly directed towards photosynthetic tissues. Fe is crucial for photosynthesis, and >80% of the Fe in mesophyll cells is concentrated in chloroplasts (Shingles *et al.*, 2002). Thus, photosynthetic tissues constitute a large Fe pool potentially available for remobilization during seed filling. However, the mechanisms of micronutrient remobilization from vegetative organs during senescence have received little attention to date (Pottier *et al.*, 2014). Senescence in *Arabidopsis thaliana* is associated with a decrease by 50% of leaf Fe concentration, in parallel with micronutrient filling in seeds (Himelblau and Amasino, 2001). This finding implies that 50% of the micronutrients present in senescent leaves are not remobilized. A better understanding of micronutrient remobilization from senescent organs is therefore likely to highlight new solutions to improve seed micronutrient content. More specifically, mechanisms controlling the availability of nutrients in source organs, such as autophagy, could be a matter of great significance for micronutrient loading in seeds (Shi *et al.*, 2012; Pottier *et al.*, 2014).

Autophagy is conserved from yeast to animals and plants. It allows degradation of cytoplasmic components by lysosomal (animals) or vacuolar (plants) internalization mediated by double membrane vesicles called autophagosomes (Reumann *et al.*, 2010). Autophagy promotes energy production, elimination of toxic components, and nutrient recycling by discarding aberrant proteins, damaged organelles, as well as normal cytoplasmic components when they are no longer useful (Yoshimoto, 2012). Autophagy-related genes (*ATG* genes) involved in autophagosome formation were first identified in yeast (Matsuura *et al.*, 1997). Subsequently, orthologues of *ATG* genes were identified in plants, and autophagy-deficient plants were characterized (Li and Vierstra, 2012). All autophagy-deficient plants exhibit hypersensitivity to carbon and nitrogen starvation, pointing to a central role for autophagy in nutrient recycling (Liu and Bassham, 2012; Guiboileau *et al.*, 2013). The up-regulation of *ATG* genes during leaf senescence in Arabidopsis suggests a role for autophagy in nutrient recycling at the end of the plant life cycle (Doelling *et al.*, 2002; van der Graaff *et al.*, 2006; Chung *et al.*, 2010; Breeze *et al.*, 2011; Liu and Bassham, 2012). In agreement with this hypothesis, nitrogen remobilization efficiency (i.e. the proportion of nitrogen allocated to seeds through remobilization from vegetative tissues during senescence) is strongly affected in autophagy-deficient plants (Guiboileau *et al.*, 2012).

In this work, we investigated the role of autophagy in metal micronutrient resorption from senescent vegetative organs to seeds in Arabidopsis. Our results indicate that micronutrient remobilization is impaired in vegetative tissues of autophagy-deficient plants. Focusing on ATG5, which is essential for autophagosome formation (Kirisako *et al.*, 2000), we show that autophagy-dependent remobilization is critical for optimal translocation of Fe as well as other metal nutrients from vegetative organs to seeds.

## Materials and methods

### Plant material and growth conditions

*Arabidopsis thaliana* (L.) ecotype Columbia-0 (Col-0) [*atg5-1* (SALK_020601), *atg9-2* (SALK_130796), AtATG18a RNAi (RNAi18), *sid2*, *atg5sid2*], and ecotype Wassilewskija (WS) [*atg4* (*atg4a4b-1*), *atg9-1*] have been described previously (Hanaoka *et al.*, 2002; Yoshimoto *et al.*, 2004, 2009; Xiong *et al.*, 2005; Inoue *et al.*, 2006).

The first experiment (experiment 1) including all autophagy-deficient plants presented in this study was conducted on sand. Seeds were sown on sand, and plants were watered three times per week for 2 h with high nitrogen nutritive solution as described in Chardon *et al.* (2010). Plants were grown under the following conditions: 8/16 h, 21/17 °C light/dark until 56 days after sowing (DAS), and then transferred to long days (16 h light), maintaining similar day/night temperatures. When plants were dry, seeds were separated from the dry remains of vegetative tissues, including leaves, stems, and siliques, to determine their respective dry weights and metal contents.

In other experiments (experiments 2 and 3) performed to determine the involvement of autophagy in Fe remobilization, plants were cultivated on a 1/1 mix of sand and perlite, and watered three times per week for 2 h with modified Hoagland solution [0.28 mM KH_2_PO_4_, 1.25 mM KNO_3_, 0.75 mM MgSO4, 1.5 mM Ca(NO_3_), 25 µM H_3_BO_3_, 50 µM KCl, 1 µM ZnSO_4_, 0.1 µM Na_2_MoO_4_, 0.5 µM CuSO_4_, 5 µM MnSO_4_, and 3 mM MES-KOH, pH 5.7]. Fe (10 µM) was provided as Fe^3+^ chelated to HBED [*N*,*N*'-di(2-hydroxybenzyl) ethylene diamine-*N*,*N*'-diacetic acid monochloride hydrate; Strem Chemicals, Newburyport, MA, USA] prepared as described by Lanquar *et al.* (2005). Plants were grown in a climate chamber under the following conditions: 75% relative humidity, 9/15 h, 21/19 °C light/dark until 68 DAS. Then, plants were transferred to long days (16 h light), maintaining similar conditions. At the onset of flowering (78 DAS), half of the plants were maintained in Fe-sufficient nutrition (solution containing 10 µM Fe-HBED) and the other half were transferred to Fe deprivation nutrition (solution containing 20 µM ferrozine) until the end of the plant life cycle. When plants were dry, leaves, seeds, and stems including empty siliques were harvested separately to determine their dry weight and their metal content. In experiment 2, light was enriched in blue and red wavelengths (OSRAM FLUORA, Munich, Germany) and light intensity was maintained to 100 µmol photons m^–2^ s^–1^. In experiment 3, which is presented in the Supplementary figures, white light was provided by TLD 58W 830 and 840 (Philips, Amsterdam, The Netherlands) with an intensity of 230 µmol photons m^–2^ s^–1^.

### Metal concentration analysis

After weight measurement, dried samples were digested in 2 ml of 70% nitric acid in a DigiBlock ED36 (LabTech, Italy) at 100 °C for 1 h, 120 °C for 6 h, and 80 °C for 1 h. After dilution in trace-metal-free water, the metal content of the samples was determined by atomic absorption spectrometry using an AA240FS flame spectrometer or with an MP AES 4200 Atomic Emission spectrometer (Agilent, USA).

### ^57^Fe labeling

For ^57^Fe labeling, ^57^Fe^3+^ (96.28 atom %) was prepared and combined with HBED to replace ^56^FeHBED by ^57^FeHBED in the modified Hoagland solution. After 4 d without watering, plants were placed in the presence of ^57^FeHBED-modified Hoagland solution for 24 h precisely, at 52 and 54 DAS, during the vegetative phase. After labeling, the pots containing substrate and roots were rinsed four times with ultrapure water and once with non-labeled solution to remove ^57^Fe. Then, unlabeled modified Hoagland solution was used for the rest of the culture cycle until harvest.

### Determination of ^57^Fe/^56^Fe ratios and remobilization indices

An aliquot of 50 mg of dried and crushed sample was digested in a mixture of nitric acid and hydrogen peroxide (ultra-pure reagent grade) on a heated DigiPrep block (SCP Science, Canada) at 90 °C for 3 h and then diluted to 50 ml with ultrapure water. Blank samples were prepared in the same conditions. The measurement of the isotopes ^56^Fe and ^57^Fe contained in these solutions was performed using inductively coupled plasma mass spectrometry (ICP-MS; Perkin Elmer Nexion, USA). Helium gas was introduced into the dynamic collision cell to prevent specific polyatomic interference caused by argon and calcium oxides. Quality control solutions were prepared from a standard solution containing 100 mg ml^–1^ of natural sourced Fe (Inorganic Ventures) and were used during the whole analytical sequence. The ^57^Fe abundance (A%) was calculated as atom percent and defined as A%=100×^57^Fe content/(^57^Fe content+^56^Fe content). The ^57^Fe abundance of unlabeled plant controls (A% _control_) was 0.0241. The ^57^Fe enrichment (E%) of samples was defined as (A% _sample_–A% _control_). The ^57^FeRSA _sample_ (relative ^57^Fe-specific allocation of the sample) was calculated as (A% _sample_–A% _control_)/(A% _labeling solution_–A% _control_). The ^57^FeRSA _seeds_/^57^FeRSA _(seeds+stems+leaves)_ ratio was calculated as (E% _seeds_)/[(E% _seeds_×Fe% _seeds_×DW _seeds_+E% _leaves_×Fe% _leaves_×DW _leaves_+ E% _stems_×Fe% _stems_×DW _stems_)/(Fe% _seeds_×DW _seeds_+Fe% _leaves_×DW _leaves_+Fe% _stems_×DW _stems_)] where E% is the ^57^Fe enrichment, Fe% is the concentration of Fe as a percentage, and DW is the dry weight (Gallais *et al.*, 2006; Masclaux-Daubresse and Chardon, 2011).

### Statistical analysis

Experiments were carried out in 3–8 independent biological replicates. Data were analyzed with Kruskal–Wallis and Mann–Whitney non-parametric tests for multiple comparisons and pair comparisons, respectively. For multiple comparisons, a Tukey post-hoc test was performed when significant differences were detected (*P*<0.05). Different letters indicate significant differences between samples. All tests were performed using the R software package.

## Results

### Autophagy-deficient plants retain higher concentrations of zinc, manganese, and iron in vegetative organs

**Fig. 1.**
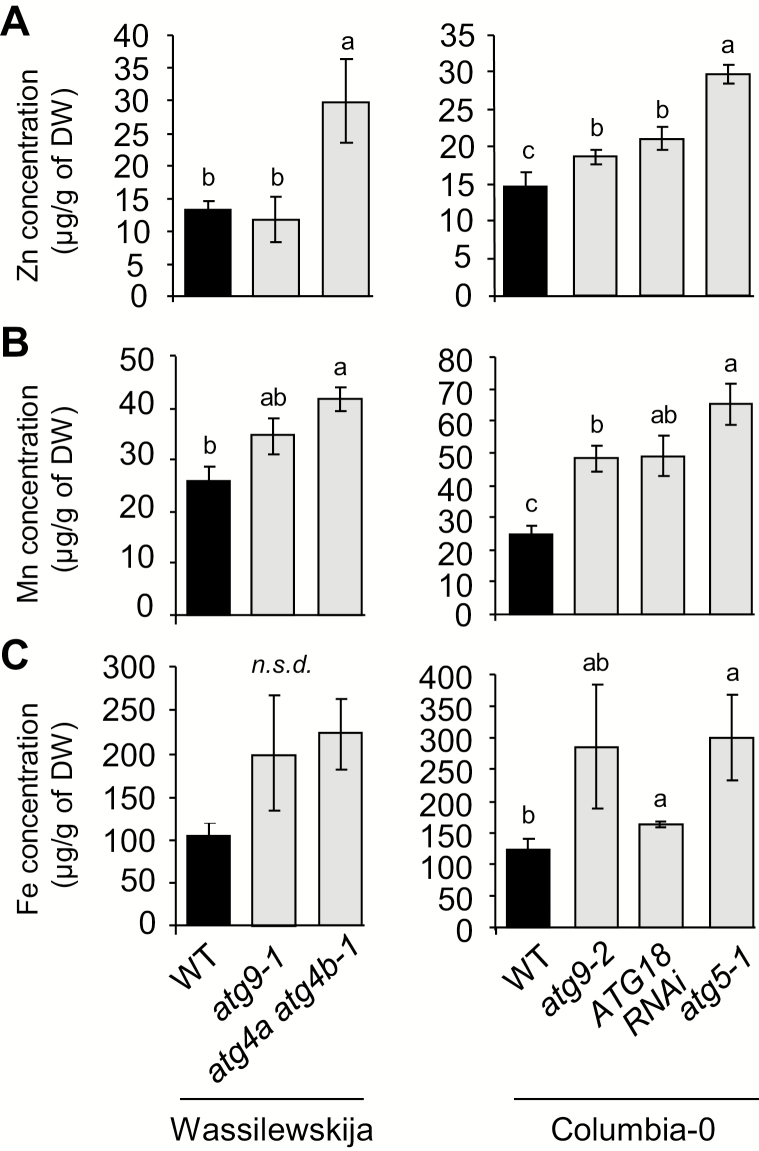
Defects in autophagy lead to elevated Zn, Mn, and Fe concentrations in vegetative tissues of *Arabidopsis thaliana* (experiment 1). Zn (A), Mn (B), and Fe (C) concentrations in dry remains of vegetative tissues of wild-type plants, (Wassilewskija and Columbia-0; black bars) and autophagy-deficient plants (gray bars) in the same genetic backgrounds, grown on sand as described in Chardon *et al.* (2010). Results are shown as the mean ±SE of four biological repeats. Different letters indicate significant differences between genotypes according to a Kruskal–Wallis test (*P*<0.05, *n*=4) followed by a Tukey post-hoc test; *n.s.d.* indicates no significant difference.

To investigate the involvement of autophagy in micronutrient remobilization from vegetative organs to seeds during senescence, we first compared metal concentrations in vegetative organs of wild-type and several autophagy-deficient plants after the completion of the plant’s life cycle in experiment 1 (Hanaoka *et al.*, 2002; Yoshimoto *et al.*, 2004; Thompson *et al.*, 2005; Xiong *et al.*, 2005; Guiboileau *et al.*, 2012). If remobilization of metal micronutrients to seeds is impaired in plants with compromised autophagy, their concentration in the dry remains of vegetative parts should be increased compared with the wild type. Manganese (Mn), Fe, and zinc (Zn) concentrations were up to 2.5 times higher in dry remains of autophagy-deficient plants compared with those of wild-type plants (Fig. 1). The highest increases in metal concentrations were consistently observed in *atg5-1* and *atg4a atg4b-1* mutants, which are fully defective in autophagosome formation (Yoshimoto *et al.*, 2004; Guiboileau *et al.*, 2012). In contrast, in dry remains of *atg9.1*, *atg9.2*, and ATG18 RNAi, increases in Mn, Fe, and Zn concentrations were smaller and, in some cases, not significant. This is consistent with the observation that autophagic bodies have been detected in *atg9.1*, *atg9.2*, and ATG18 RNAi plants, indicating that autophagy is not fully compromised in these mutants (Yoshimoto *et al.*, 2004; Guiboileau *et al.*, 2012).

These data suggest that defects in autophagy affect micronutrient metal resorption from vegetative organs. The severity of the resorption defects appears to be related to the level of autophagy deficiency in the different genotypes tested.

### Distinct mechanisms lead to decrease in Fe concentrations in seeds of autophagy-deficient plants depending on Fe supply during the reproductive stage

To analyze further the role of autophagy in micronutrient resorption from vegetative organs to seeds, we chose to focus on the *atg5-1* mutant, which exhibited robust increases in metal concentrations in its dry remains (Fig. 1), and on Fe, a critical micronutrient that is often not sufficiently available in seeds of crop plants for human nutrition (Velu *et al.*, 2014). Because autophagy operates a feedback loop modulating salicylic acid (SA) signaling to regulate senescence negatively, autophagy-deficient plants exhibit a premature senescence phenotype which may interfere with nutrient resorption (Yoshimoto *et al.*, 2009; Guiboileau *et al.*, 2012). Hence, we also included in this experiment (experiment 2) the stay-green mutant *sid2*, which is defective in SA biosynthesis, and the *atg5sid2* double mutant, in which autophagy is defective while senescence is delayed (Wildemuth *et al*., 2001; Yoshimoto *et al.*, 2009).

Seed filling may depend on both remobilization from senescent organs and *de novo* uptake by roots during seed formation. In order to discriminate between the two pathways, plants were grown in parallel under two different conditions: some were supplied with sufficient Fe during their whole life cycle (Fe sufficient), while other were deprived of Fe from the onset of flowering (Fe deprivation).

**Fig. 2.**
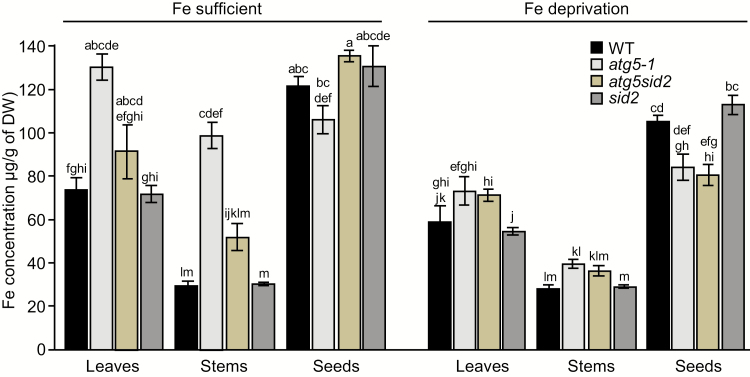
Distinct mechanisms lead to a decrease in Fe concentration in seeds of autophagy-deficient plants depending on Fe supply during the reproductive stage in *Arabidopsis thaliana* (experiment 2). Fe concentrations in leaves, stems (including empty siliques), and seeds of wild-type (WT, black bars), *atg5-1* mutant (light gray bars), *atg5sid2* double mutant (intermediate gray bars), and *sid2* (dark gray bars) mutant plants grown on sand/perlite (1/1) substrate watered with modified Hoagland medium. Iron was supplied (Fe sufficient) or not (Fe deprivation) during the reproductive stage. Results are shown as the mean ±SE of five biological repeats. Different letters indicate significant differences according to a Kruskal–Wallis test (*P*<0.05, *n*=5) followed by a Tukey post-hoc test.

Before investigating the contribution of autophagy to Fe remobilization from different organs and subsequent Fe loading in seeds during the reproductive stage, Fe concentrations were first measured in roots and rosette leaves during the vegetative growth of wild-type and *atg5-1* mutant plants (Supplementary Fig. S1, experiment 2, at *JXB* online). No significant difference in Fe concentration was observed between genotypes, indicating that Fe uptake and transfer to leaves was not affected in *atg5-1* at this stage (Supplementary Fig. S1). A 3.2 ± 0.6-fold increase in the level of ferritins (*n*=4; *P*<0.05 according to a Mann–Whitney test; Supplementary Fig. S2), which are involved in Fe storage in plastids (Briat *et al.*, 2010), was observed at the vegetative stage in *atg5-1* mutant leaves compared with those of wild-type plants. This result suggests that the Fe subcellular distribution is modified in *atg5-1*. Fe concentrations were then measured in leaves, stems including empty siliques, and seeds of *atg5-1*, *atg5sid2*, and *sid2* mutants, and wild-type plants at the end of their life cycle. No differences in Fe concentration were observed between *sid2* and wild-type plants, irrespective of the Fe nutrition regime. However, under Fe-sufficient nutrition, Fe concentrations in the *atg5-1* mutant were significantly higher than those of the wild type in leaves (1.8-fold) and in stems (3.4-fold) (Fig. 2), in agreement with the results presented in Fig. 1. The increase in Fe concentration in *atg5-1* vegetative organs was associated with a trend towards decreased seed Fe concentration (Fig. 2). Similar results were observed in experiment 3 comparing wild-type and *atg5-1* mutant plants under higher light conditions (Supplementary Fig. S3), confirming the robustness of this observation. In experiment 3, metal concentrations were also measured in roots and rosette leaves at the beginning of seed filling (Supplementary Fig. S4). Under Fe-sufficient conditions, higher Fe concentrations were observed in rosette leaves of the *atg5-1* mutant than in those of wild-type plants already at this early stage, whereas no difference was detected at the root level (Supplementary Fig. S4). These data suggest that, at least initially, Fe is remobilized from leaves rather than from roots. Both the increase in Fe concentration in vegetative organs and the decrease in seeds observed in *atg5-1* tended to be reversed to a large extent in *atg5sid2* (Fig. 2, experiment 2), suggesting that these effects are in part due to early leaf senescence.

Under Fe deprivation, Fe concentrations were lower in leaves and seeds irrespective of the genotype, as expected (Fig. 2). In contrast to the Fe-sufficient nutrition, Fe concentrations in vegetative organs of the *atg5-1* mutant displayed only a moderate and non-significant increase (1.2-fold) with respect to the wild type, while Fe concentration was significantly decreased by 20% in seeds (Fig. 2). These effects were not reversed in the *atg5sid2* mutant, suggesting that they are due to impaired autophagy rather than early senescence.

Taken together, these data indicate that the *atg5-1* mutant is affected in Fe resorption from vegetative organs to seeds by distinct mechanisms under sufficient Fe supply or Fe deprivation during the reproductive period.

### Defective autophagy leads to a strong decrease in the pool of Fe allocated to seeds

To evaluate the impact of autophagy on the redistribution of Fe pools between vegetative organs and seeds, we calculated Fe mass distribution among organs. For this purpose, we first determined the total biomass distribution as well as the harvest index, which is defined as the ratio of seed biomass relative to the whole aerial biomass of the plant after completion of the life cycle. As previously reported (Guiboileau *et al.*, 2012), loss of *ATG5* function led to a strong reduction of total dry biomass (2.8-fold; Supplementary Fig. S5) and an even more severe defect in seed production causing a decrease in the harvest index (Supplementary Fig. S6). These effects were not significantly reversed in the *atg5sid2* mutant, and were also observed when plants were deprived of Fe during the reproductive phase (Supplementary Figs S5, S6).

**Fig. 3.**
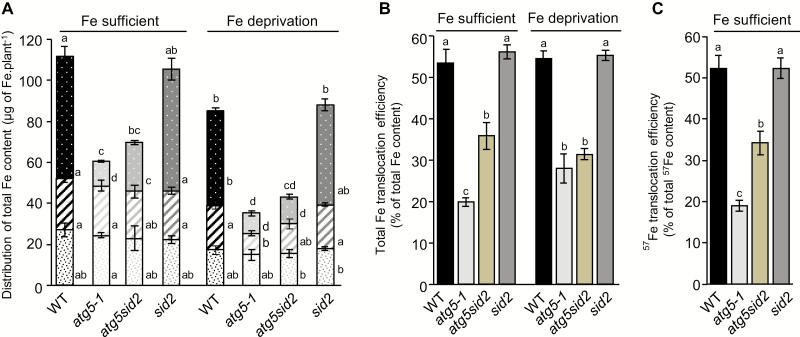
Distribution of total Fe content and Fe translocation to seeds are dramatically affected in *atg5-1* and *atg5sid2* mutants of *Arabidopsis thaliana* (experiment 2). (A) Distribution of Fe between leaves (filled columns), stems including empty siliques (diagonal stripes), and seeds (white dots) of plants are represented for the wild-type (WT, black bars), the *atg5-1* (light gray bars), the *atg5sid2* (intermediate gray bars), and the *sid2* (dark gray bars) mutant plants. (B) Total Fe translocation efficiency calculated as the ratio between the Fe content in seeds and the Fe content in the whole plant (Fe _seed_/(Fe _leaf_+Fe _stem_+Fe _seed_). (C) ^57^Fe translocation efficiency (remobilized Fe translocation efficiency) calculated as ^57^Fe_seed_/(^57^Fe _leaf_+^57^Fe _stem_+^57^Fe _seed_) after ^57^Fe pulse labeling at the vegetative stage. Plants were grown on sand/perlite (1/1) substrate watered with modified Hoagland medium. Fe was supplied (Fe sufficient) or not (Fe deprivation) during the reproductive stage. Results are shown as the mean ±SE of five biological repeats. Different letters indicate significant differences between genotypes and conditions according to a Kruskal–Wallis test (*P*<0.05, *n*=5) followed by a Tukey post-hoc test.

Taking biomass and Fe concentrations into account, Fe content [Fe_content_ (µg)=Fe_concentration_ (µg mg^–1^ of DW)×Organ_biomass_ (mg DW)] was determined for each organ (Fig. 3A). First, we observed a lower Fe content under Fe deprivation in all genotypes, indicating that *de novo* Fe uptake during seed formation contributes to the total Fe content independently of autophagy (Fig. 3A). Although the *atg5-1* mutant displayed significantly higher Fe concentrations than the wild type in vegetative organs (Fig. 2), its total Fe content was strongly decreased compared with the wild type due to its much lower biomass (Fig. 3A; Supplementary Fig. S5). Disrupting the *sid2* gene did not restore Fe accumulation in *atg5-1*, indicating that this defect is not due to the premature senescence of the *atg5-1* mutant.

Compared with that of the wild type, lower Fe contents were observed in vegetative organs of *atg5-1* (–35%) and *atg5sid2* (–24%) mutants specifically under Fe deprivation but not under Fe-sufficient nutrition (Fig. 3A). This result indicates that Fe supply during the reproductive stage provides vegetative organs of autophagy-deficient plants with Fe even though they undergo premature leaf senescence. This suggests that Fe is accumulated in vegetative tissues during the early steps of the reproductive stage.

The Fe content of seeds from plants growing under Fe deprivation was dramatically decreased by –80% and –70% in *atg5-1* and *atg5sid2* mutants compared with the wild type, respectively (Fig. 3A). Fe content is therefore much more severely affected by the lack of autophagy in seeds than in other organs due to a cumulative effect of decreased seed production and decreased seed Fe concentration. Fe supply during the reproductive stage partially restored the Fe content of *atg5sid2* seeds but had no effect on the Fe content of *atg5-1* seeds. This result indicates that *de novo* Fe uptake during seed formation can provide seeds with Fe in autophagy-deficient plants, but only when premature senescence is prevented. This suggests that such a contribution takes place during the late steps of the reproductive stage, when *atg5-1* but not *atg5sid2* displays leaf senescence symptoms.

### Impaired autophagy affects translocation of Fe remobilized from vegetative organs to seeds

We then analyzed the impact of the absence of autophagy on the efficiency of total Fe translocation to seeds (Fe content _seeds_/Fe content _(leaves+stems+seeds)_), under Fe-sufficient nutrition and Fe deprivation (Fig. 3B). The efficiency of total Fe translocation to seeds was indistinguishable between wild-type and *sid2* mutant plants, representing 50–55% of the total Fe content of the plant, irrespective of the Fe nutrition regime. In contrast, the efficiency of total Fe translocation was strongly decreased in *atg5-1* and *atg5sid2* mutants. Under Fe-sufficient nutrition, Fe translocation efficiency was decreased by 63% in the *atg5-1* mutant and by only 35% in the *atg5sid2* mutant, confirming that both autophagy and premature senescence limit Fe translocation to seeds under these conditions. Under Fe deprivation, total Fe translocation efficiency was decreased to a similar extent (42–48%) in *atg5-1* and *atg5sid2* mutants, indicating that premature senescence has no impact on total Fe translocation when Fe is not provided during the reproductive phase.

In experiment 3 performed under higher light conditions, similar total Fe translocation efficiencies of 50–55% were measured in the wild type (Supplementary Fig. S7). However, in the *atg5-1* mutant, the total Fe translocation efficiency was further decreased under Fe deprivation. We hypothesize that light stress may have modified the timing of leaf senescence or amplified the effect of Fe deficiency.

Using Fe deprivation to estimate the relative contribution of root uptake and remobilization from leaves may be biased by the activation of compensatory mechanisms under Fe deficiency (Pottier *et al.*, 2014). Then, to monitor fluxes specifically between senescing organs and seeds independently of uptake from soil, we performed a ^57^Fe labeling pulse–chase experiment. A pulse of ^57^Fe was provided in the nutritive medium of young rosette plants. Pots and substrate were then thoroughly washed to avoid further ^57^Fe uptake during the chase period. Plants were subsequently watered with nutritive solution containing non-labeled Fe until the end of the life cycle (Fe-sufficient nutrition). At the end of the life cycle, the relative abundance of ^57^Fe was determined in each organ by ICP-MS. From these data, we first calculated ^57^FeRSA values which reflect the dilution of ^57^Fe in leaves, stems, and seeds for each genotype (Supplementay Table S1). Leaves displayed the highest ^57^FeRSA values (Supplementary Table S1), which is consistent with the fact that pulse labeling was performed at the rosette stage when other organs were not yet present. Conversely, stems showed consistently the lowest ^57^FeRSA, suggesting that both Fe remobilization from rosette leaves and *de novo* Fe uptake from soil contributed to stem total Fe (Supplementary Table S1). Finally seeds displayed an intermediate ^57^FeRSA level which could reflect a mixed contribution of remobilization from leaves and stems, and possibly *de novo* Fe uptake during the reproductive stage. The lower ^57^FeRSA in *sid2* and *atg5sid2* seeds may be due to delayed senescence allowing prolonged uptake of ^56^Fe and further dilution of the label. To obtain further information on the contribution of these different sources of Fe to seed Fe, we calculated the ^57^FeRSA _seeds_/^57^FeRSA _(seeds+stems+leaves)_ ratio (Table 1), which compares ^57^Fe dilution between the seeds and the whole aerial part of the plant (Gallais *et al.*, 2006). A ^57^FeRSA _seeds_/^57^ FeRSA_(seeds+stems+leaves)_ ratio lower than 1 indicates that the ^56^Fe absorbed *de novo* during the reproductive stage contributes mainly to seed Fe content, while a ratio higher than 1 indicates that the ^56^Fe absorbed during the reproductive stage contributes mainly to the Fe content of vegetative tissues. Here, ^57^FeRSA _seeds_/ ^57^FeRSA_(seeds+stems+leaves)_ ratios were almost equal to 1, indicating that ^57^Fe dilution is similar in seeds and in the whole plant, and no significant differences were observed between genotypes (Table 1). Such a result shows that ^56^Fe taken up during the reproductive stage contributes to a similar extent to Fe content of seeds and to that of the leaves and stems. Two scenarios may account for this value, either (i) direct ^56^Fe fluxes to the vegetative organs and to the seeds are perfectly balanced and lead to similar dilutions or (ii) ^56^Fe is first loaded in vegetative organs, which subsequently provide Fe with a pre-determined RSA to the seeds. Finally, combining the ^57^Fe/^56^Fe ratio with Fe concentration and DW, we calculated the ^57^Fe content_seed_/ ^57^Fe content_(leaves+stems+seeds)_ ratio (i.e. the proportion of ^57^Fe translocated from the rosettes and the stems to the seeds). Compared with the apparent Fe translocation ratio, this ratio takes into account Fe taken up only during the vegetative stage and is thus a good indicator of the Fe remobilization efficiency from vegetative organs to seeds even when Fe is supplied during the reproductive stage (Fig. 3C). Values of the ^57^Fe content _seed_/ ^57^Fe content_(leaves+stems+seeds)_ ratio were very similar to those of the Fe content_seed_/Fe content_(leaves+stems+seeds)_ ratio (Fig. 3B) irrespective of the genotype. This result suggests that, under our experimental conditions, the loading of Fe to the seeds is mostly achieved by Fe remobilization from the vegetative organs according to scenario (ii), even though Fe is still taken up from the soil during the reproductive stage (Fig. 3A). Thus, the intermediate ^57^FeRSA level measured in seeds is only due to the mixed contribution of remobilization from vegetative organs (Supplementary Table S1) rather than to dilution by ^56^Fe originating directly from root uptake. This is in apparent contradiction to a value of ^57^FeRSA _seeds_/^57^FeRSA _(seeds+stems+leaves)_ of 1, indicating that *de novo* uptake during the reproductive growth contributes similarly to Fe content of vegetative organs and seeds (Table 1). This apparent discrepancy may be solved if we consider scenario (ii) which suggests an indirect involvement of the *de novo* Fe uptake to seed Fe content though a two-step mechanism, as described in the Discussion.

**Table 1.** ^57^FeRSA _seeds_ /^57^FeRSA _(seeds+stems+leaves)_ ratios in wild-type Columbia-0, *atg5-1*, *atg5sid2*, and *sid2* mutant plants which have undergone ^57^Fe pulse labeling at the vegetative stage

Genotype	^57^FeRSA _seeds_/^57^FeRSA _(seeds+stems+leaves)_
Col-0	0.96 ± 0.02
*atg5-1*	0.98 ± 0.07
*atg5sid2*	0.92 ± 0.04
*sid2*	0.88 ± 0.04

No significant differences in ^57^FeRSA _seeds_ /^57^FeRSA _(seeds + stems + leaves)_ ratios were observed between genotypes according to Kruskal–Wallis test (*P*<0.05, *n*=5).

### The lack of autophagy also impacts Zn and Mn translocation to seeds

Before investigating the putative involvement of autophagy in the translocation of Zn and Mn to seeds, their concentrations were measured in roots and young rosette leaves during the vegetative stage, to test whether *atg5-1* affects Mn or Zn uptake and transfer to leaves at this stage (Supplementary Fig. S1). For Zn, no significant differences were observed in leaves and roots, as was the case for Fe, indicating similar Zn uptake in both genotypes (Supplementary Fig. S1). In experiment 3, metal concentrations were also measured in roots and rosette leaves at the beginning of seed filling (Supplementary Fig. S4). At this developmental stage, the Zn concentration was significantly higher in both roots and leaves of the *atg5-1* mutant than in those of wild-type plants (Supplementary Fig. S4). Such a result suggests therefore that unlike Fe, autophagy-dependent Zn remobilization during seed loading originates from both roots and leaves. The Mn concentration in roots was more than twice as high in the *atg5-1* mutant compared with wild-type plants at the vegetative stage (Supplementary Fig. S1, experiment 2). In experiment 3, root Mn concentration measured at the beginning of seed loading was also much higher in *atg5-1* (Supplementary Fig. S4). These observations indicate that disruption of the *ATG5* gene strongly perturbs Mn uptake and/or Mn remobilization from roots during both vegetative and reproductive growth.

**Fig. 4.**
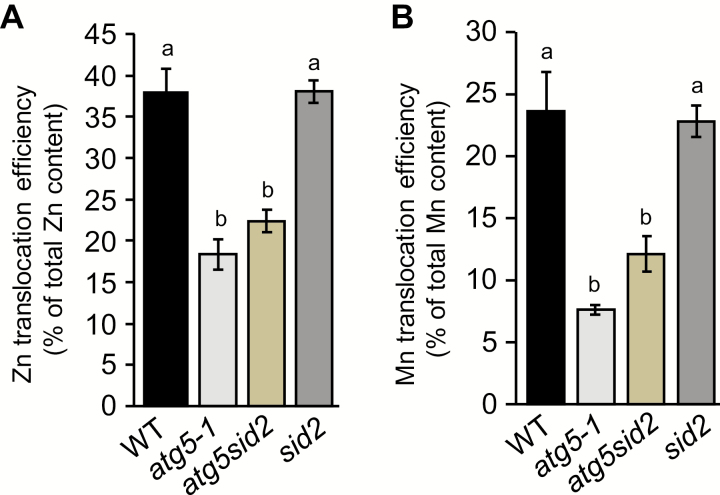
Translocation efficiencies of Zn (A) and Mn (B) are also affected in *atg5-1* and *atg5sid2* mutant plants of *Arabidopsis thaliana* (experiment 2). Translocation efficiencies were calculated for wild-type (WT), *atg5-1*, *atg5sid2*, and *sid2* mutant plants, as the ratio between the micronutrient content in seeds and the micronutrient content in the whole plant. Plants were grown on sand/perlite (1/1) substrate watered with modified Hoagland medium containing Fe during the whole plant cycle. Results are shown as the mean ±SE of five biological replicates. Different letters indicate significant differences between genotypes according to a Kruskal–Wallis test (*P*<0.05, *n*=5) followed by a Tukey post-hoc test.

Concentrations were studied at the end of the plant life cycle in order to determine Mn and Zn translocation efficiencies under Fe-sufficient nutrition, following the same approach as for Fe. In wild-type plants, Zn and Mn translocation efficiencies were 38% and 24%, respectively (Fig. 4). In *atg5-1* mutants, total translocation efficiencies of these elements were both strongly affected with respect to the wild type. Similar results were observed in experiment 3 performed under higher light conditions (Supplementary Fig. S7). Also, no significant differences were observed between *atg5-1* and *atg5sid2* mutant plants (Fig. 4). Thus, a defect in autophagy affects not only Fe translocation but also, to various extents, translocation of other micronutrients, even in the absence of premature senescence. In view of the results obtained at earlier stages (Supplementary Figs S1, S4), remobilization from roots should be taken into account to evaluate fully the importance of autophagy for Zn and especially for Mn translocation to seeds. The ratios calculated on the basis of leaf and stem Mn and Zn content (Fig. 4) probably underestimate the effect of impairing autophagy.

## Discussion

Because of its poor solubility in most soils, its essential roles in plant cellular processes, and due to its insufficiency in the human diet, many investigations have been undertaken during the last decades to decipher the route of Fe within the plant from its uptake from the rhizosphere to its loading into seeds. Both uptake from soil and remobilization from senescent organs participate in grain loading and thereby contribute to Fe use efficiency (Pottier *et al.*, 2014). To date, little is known about the mechanisms involved in Fe remobilization from senescent source organs during seed formation. A first step of disassembling of organelles, proteins, and various macromolecules is required prior to nutrient reallocation. During senescence, numerous genes involved in catabolism and degradation mechanisms including genes involved in autophagy are up-regulated following a well-established schedule (Breeze *et al.*, 2011). Thus, autophagy may be a limiting step in making nutrients available for further reallocation towards new organs. In the present work, we have investigated the involvement of autophagy in metal micronutrient remobilization from vegetative organs to seeds. Analyzing a range of autophagy mutants, we found that the defect in nutrient remobilization correlated with the severity of the defect in autophagosome formation (Fig. 1). Accordingly, the severe defect in autophagy in the *atg5-1* mutant leads to a decrease in seed Fe concentration and a drastic reduction in Fe translocation efficiency to seeds (Figs 2, 3; Supplementary Fig. S3). Interestingly, this defect could be alleviated by providing Fe to the roots only when *atg5-1* premature senescence was prevented (Fig. 3A). These results, together with the outcome of the ^57^Fe pulse labeling experiment, suggest that the ^56^Fe taken up by the roots during seed formation contributes indirectly to seed Fe content (Table 1; Fig. 3B, C). Finally, we found that autophagy plays roles not only in Fe but also in Zn and Mn translocation to seeds, supporting the importance of this mechanism for optimal loading of mineral nutrients to seeds (Fig. 4).

### Mineral translocation efficiency to seeds is constant in Arabidopsis

Total iron translocation efficiency of *A. thaliana* Columbia-0 wild-type was within the 50–55% range irrespective of light conditions or Fe nutrition regime (Fig. 3B; Supplemental Fig. S5). Fe uptake observed during the reproductive stage under Fe-sufficient nutrition therefore has no impact on Fe translocation efficiency of wild-type plants (Fig. 3A, B). A previous study also found that half of the Fe content of Arabidopsis leaves was remobilized during senescence (Himelblau and Amasino, 2001), even though the ecotype, the photoperiod, and the growth conditions were different. Thus, Fe translocation efficiency appears to be remarkably stable in *A. thaliana*. The other micronutrients studied in this work exhibited lower total translocation efficiencies than Fe (Fig. 4; Supplementary Fig. S7). We found, however, that Zn is better translocated than Mn, which is also in agreement with results obtained by Himelblau and Amasino (2001). In contrast, Maillard *et al.* (2015) found large variation in leaf mineral nutrient resorption between different species. Pottier *et al.* (2015*a*) also found variation in metal resorption from leaves from different poplar genotypes. However, remobilization from leaves rather than translocation to seeds was analyzed in these studies.

### Seed iron first transits through vegetative organs

**Fig. 5.**
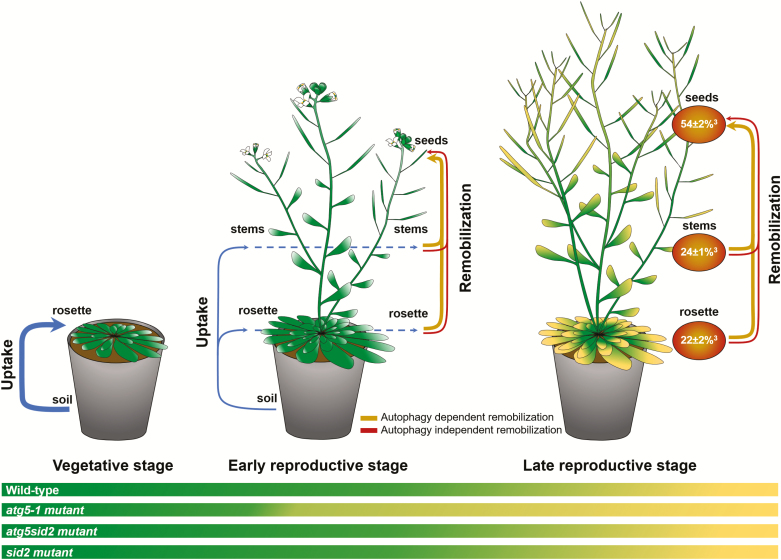
Iron fluxes in wild-type *Arabidopsis thaliana* growing under Fe-sufficient conditions, in vegetative, early reproductive, and late reproductive stages, based on results obtained in wild-type and autophagy-deficient plants. Thicknesses of the uptake and remobilization arrows are proportional to fluxes. Fe distribution (brown circles) is expressed as a percentage of total Fe of wild-type plants harvested after completion of the life cycle. Early reproductive stage is defined as the period of premature senescence in the *atg5-1* mutant. Late reproductive stage is defined as the period of senescence in the wild type, when the *atg5-1* mutant is already dry. Together, the results obtained indicate that (i) most of the Fe uptake (71 ± 4%^1^) takes place during the vegetative growth; (ii) Fe uptake after the onset of flowering (29 ± 5%^2^) occurs mostly during the early reproductive stage; (iii) Fe loading into seeds (54 ± 2%^3^) is mainly achieved through remobilization rather than a direct contribution from Fe root uptake; and (iv) in the wild type, 35 ± 2%^4^ of the total Fe content is loaded to seeds through autophagy-dependent remobilization, and 19 ± 2%^5^ is loaded through autophagy independent remobilization. ^1^Total Fe content of plants growing in Fe deprivation conditions as a percentage of total iron of plants growing in Fe-sufficient conditions. ^2^Difference between the total Fe content of plants growing in Fe-sufficient conditions and the total Fe content of plants growing in Fe deprivation conditions as a percentage of total Fe of plants growing in Fe-sufficient conditions. ^3^Fe content in seeds, stems, and rosette leaves of wild-type plants as percentages of total Fe of wild-type plants growing in Fe-sufficient conditions. ^4^Difference between the percentage of Fe loaded in seeds of wild-type plants and the percentage of Fe loaded in seeds of *atg5sid2* mutant plants. ^5^Difference between the percentage of Fe content in seeds and the percentage of Fe loaded in seeds through autophagy-dependent remobilization.

The Fe that is taken up *de novo* during the reproductive stage may be loaded first in young parts of the vegetative organs or directly into seeds. The *atg5-1* mutant, which undergoes premature senescence, displayed a similar increase of total Fe content to that in the other genotypes when Fe is provided during the reproductive stage (Fig. 3A). This observation indicates that Fe uptake after the onset of flowering occurs essentially during the early period of the reproductive stage, prior to the senescence of *atg5-1* mutant leaves. Moreover, the *atg5-1* mutant accumulated *de novo* absorbed Fe preferentially in vegetative organs while it was translocated more efficiently to seeds when premature senescence was prevented (Fig. 3A). If senescence, which eventually leads to cell death, takes place too early, the lapse of time during which nutrients can be translocated between leaves and seeds is shortened, resulting in decreased nutrient translocation efficiency. These results suggest a two-stage process in which Fe taken up by roots is transiently accumulated in vegetative organs prior to its remobilization to the seeds, as illustrated in Fig. 5. This hypothesis also accounts for the similar values observed for total Fe and ^57^Fe remobilization efficiencies as well as for the similar ^57^Fe dilution in seeds and vegetative organs (leaves and stems) reflected in the ^57^FeRSA_seeds_/ ^57^FeRSA _(seeds+stems+leaves)_ ratio almost equal to 1 observed in all genotypes after pulse labeling during the vegetative phase (Fig. 3B, C; Table 1). Waters and Grusak (2008) concluded that *de novo* uptake is at least as important as remobilization for seed filling. However, they were using different conditions and did not consider the possibility that minerals have to transit to vegetative organs before being translocated to seeds.

### A possible contribution of root metal micronutrient stores to seed filling

Our work has not addressed the contribution of root Fe stores to total plant Fe and its fate during senescence. The experimental set up that was chosen, growth in a sand–perlite mixture, precluded harvesting the whole root system at the end of the plant’s life cycle. Moreover, rigorous analysis of root micronutrient content requires the removal through desorption of cations that are adsorbed on the root but not taken up by living cells. However, at the end of the life cycle, the roots are dead, cellular ion content is released, and desorption becomes meaningless. Growing plants under hydroponic conditions would allow collection and desorption of the entire root biomass. However, this cultivation system affects nutrient distribution between roots and shoots, and is not suitable for seed production (Diaz *et al.*, 2008).

Nevertheless, we collected roots at earlier stages of plant development. Root Fe concentrations were not affected in the *atg5-1* mutant at the vegetative stage or at the onset of silique development (Supplementary Figs S1, S4). This suggests that the defects in Fe distribution observed in the aerial parts of *atg5-1* are not related to root function. Roots are the site of *de novo* Fe uptake as well as a vegetative organ in which Fe may be stored for subsequent remobilization. The results obtained using ^57^Fe labeling argue in favor of a model in which the Fe taken up by roots during the reproductive phase first transits through vegetative organs. However, these results do not exclude a contribution of the Fe stored in roots to seed filling as well as an impact of autophagy defects on remobilization of root Fe pools at later stages. In contrast, differences in Zn concentrations in both rosette leaves and roots at the onset of seed filling suggest that root Zn pools may be reallocated together with leaf Zn pools at the reproductive stage (Supplementary Fig. S4). In addition, differences in Mn concentrations were observed in roots at the vegetative stage and at the early reproductive stage, indicating that autophagy influences its uptake or distribution (Supplementary Figs S1, S4). The importance of root Mn pools for Mn redistribution has already been documented in *Brassica napus* subjected to Mn deficiency (Maillard *et al.*, 2015). Regarding nitrogen, remobilization from roots to flowering stems and reproductive organs was shown to make a minor contribution in *A. thaliana* (Diaz *et al.*, 2008). In *B. napus*, such remobilization contributes only to 11.1% of the pod nitrogen content (Rossato *et al.*, 2001). Regarding Zn, no decrease of its root content was observed between anthesis and maturity in rice (Jiang *et al.*, 2008), while, in wheat, it was decreased by 50%, indicating that remobilization of Zn from roots varies according to species (Kutman *et al.*, 2012). In *A. thaliana*, to our knowledge, no evidence for micronutrient remobilization from roots has been reported. Also, root senescence related to seed loading still has to be investigated (Hammond and White, 2008; Lynch and Brown, 2008). Even though roots were shown to contain higher Fe concentrations than shoots in *A. thaliana* (Cassin *et al.*, 2009; Reyt *et al.*, 2015; Pottier *et al.*, 2015*b*; Eroglu *et al.*, 2016; Li *et al.*, 2016; this study), their contribution to seed filling remains to be demonstrated.

### Harnessing autophagy to improve seed nutrient content

We found that autophagy is a crucial component of micronutrient filling to seeds and thus a strong determinant of seed quality. Although autophagy was shown to be induced during senescence (van der Graaff *et al.*, 2006; Breeze *et al.*, 2011; Htwe *et al.*, 2011), significant amounts of nutrients present in vegetative organs, 30% of N in optimal conditions, 46% of Fe, and even larger proportions of other micronutrients, are not reallocated to seeds (Fig. 4; Guiboileau *et al.*, 2012). Thus, increasing the autophagy process further specifically during seed filling may be a powerful approach to increase the pool of nutrients available for subsequent translocation to seeds. However, it will also be necessary to increase seed storage capacity. In seeds, Fe is highly concentrated in vacuoles of the aleurone layer in cereals (Moore *et al.*, 2012) and of the proto-endodermis in several dicot species (Mary *et al.*, 2015; Ibeas *et al.*, 2017). These locations probably minimize the damage that could be caused by excess free Fe. Additional parallel research on the mechanisms of Fe storage in seeds is also needed. Moreover, autophagy is a complex mechanism subjected to a subtle multiscale regulation (Yoshimoto, 2012; Masclaux-Daubresse *et al.*, 2017). Further investigations on the regulation of autophagy may thus help improve grain yield by bioengineering strategies or marker-assisted breeding.

## Supplementary data

Supplementary data are available at *JXB* online.

**Fig. S1.** Fe, Mn, and Zn concentrations in roots and rosette leaves measured during vegetative growth in Col-0 wild type and the *atg5-1* mutant (experiment 2).

**Fig. S2.** Ferritins are more abundant in vegetative tissues of the *atg5-1* mutant than in those of Col-0 wild type (experiment 1).

**Fig. S3.** The increase in Fe concentration in leaves and stems of the *atg5-1* mutant is associated with a significant decrease in seed Fe concentration (experiment 3).

**Fig. S4.** Fe, Mn, and Zn concentrations in roots and rosette leaves measured at the onset of seed loading in Col-0 wild type and the *atg5-1* mutant (experiment 3).

**Fig. S5.** Total dry biomass and dry biomass distribution between organs are dramatically affected in *atg5-1* and *atg5sid2* mutants, even under Fe-sufficient conditions (experiment 2).

**Fig. S6.** Harvest index is affected in autophagy-deficient plants even in the absence of premature leaf senescence, independently of the Fe supply conditions (experiment 2).

**Fig. S7.** Total seed production and total iron translocation efficiency are dramatically affected in the *atg5-1* mutant even under Fe-sufficient conditions (experiment 3).

**Fig. S8.** Translocation efficiencies of Zn (A) and Mn (B) are affected in the *atg5-1* mutant (experiment 3).

**Table S1.** Relative ^57^Fe-specific allocation (^57^FeRSA) in leaves, stems including empty siliques, and seeds in wild-type Co-0, *atg5-1*, *atg5sid2*, and *sid2* mutant plants which have undergone a ^57^Fe pulse labeling at the vegetative stage (experiment 2).

Supplementary Table

Supplementary Figure
