# Supplementary material for: Autophagy is essential for optimal translocation of iron to seeds in Arabidopsis
Source: J Exp Bot. 2018 Nov 4;70(3):859–69. doi: 10.1093/jxb/ery388 (PMC6363094; doi:10.1093/jxb/ery388)
Supplement: Supplementary Table [file ery388_suppl_supplementary_table.pdf]

Supplemental table S1. Relative ^{57}Fe-specific allocation (^{57}FeRSA) in leaves, stems including empty siliques, seeds in wild-type Columbia-0, *atg5-1*, *atg5sid2* and *sid2* mutant plants which have undergone a ^{57}Fe pulse labeling at the vegetative stage.
Different letters indicate significant ^{57}FeRSA differences between organs and genotypes according to Kruskal-Wallis test ($p < 0.05$, n = 5) followed by a Tukey *post hoc* test.

Genotype	^{57}FeRSA seeds	^{57}FeRSA stems	^{57}FeRSA leaves
Col-0	0.051 ± 0.001^{b}	0.043 ± 0.002^{bc}	0.067 ± 0.002^{a}
atg5-1	0.050 ± 0.003^{ab}	0.029 ± 0.003^{c}	0.072 ± 0.003^{a}
atg5sid2	0.035 ± 0.002^{cd}	0.027 ± 0.001^{d}	0.053 ± 0.001^{b}
sid2	0.036 ± 0.002^{c}	0.034 ± 0.003^{c}	0.061 ± 0.005^{ab}
