# Supplementary material for: Autophagy is essential for optimal translocation of iron to seeds in Arabidopsis
Source: J Exp Bot. 2018 Nov 4;70(3):859–69. doi: 10.1093/jxb/ery388 (PMC6363094; doi:10.1093/jxb/ery388)
Supplement: Supplementary Figure [file ery388_suppl_supplementary_figure.pdf]

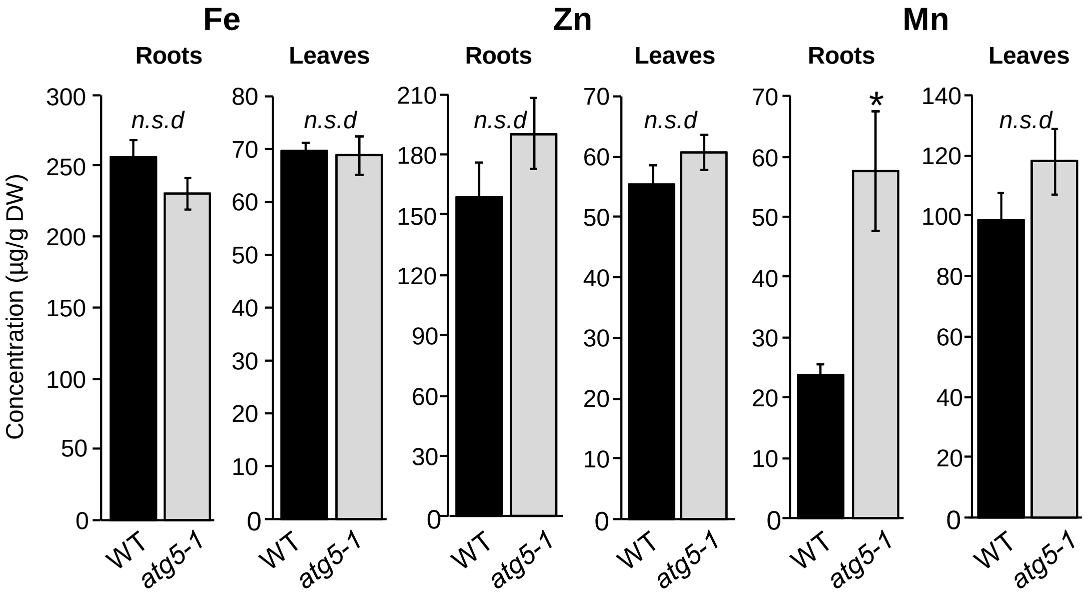

Supplemental figure S1. Fe, Zn, and Mn concentrations in roots and rosette leaves measured during the vegetative growth in wild-type (WT, black bars) and *atg5-1* mutant (gray bars) *Arabidopsis* (experiment 2). Plants were grown on sand/perlite (1/1) substrate watered with modified Hoagland medium supplemented with 10 µM FeHBED. Results are shown as means of eight biological repeats ± SE. * indicate significant differences between wild-type and *atg5-1* plants according to a Mann-Whitney test ($p < 0.05$, n = 8). *n.s.d* : no significant difference.

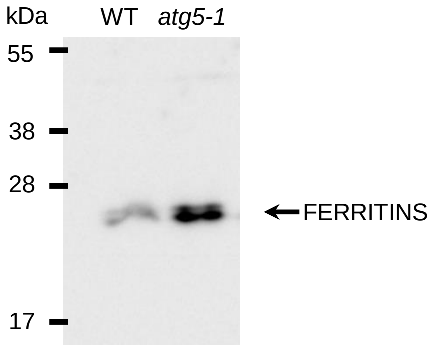

Supplemental figure S2. FERRITINS are more abundant in vegetative tissues of *atg5-1* mutant than in those of Col-0 wild-type (WT) *Arabidopsis*. A representative experiment out of 4 is shown. Total protein extract (10 µg) from 30 days-old *Arabidopsis thaliana* plants growing on sand as described in Chardon *et al.* (2010), were analyzed by SDS-PAGE and western blotting. Briefly, proteins were subjected to electrophoresis on a 13% polyacrylamide/0.1% SDS gel by the method of Laemmli. After electroblotting on to Hybond-P membrane (Amersham Biosciences), immunodetection of ferritin was performed by using a rabbit polyclonal antiserum raised against purified AtFer1 protein (1/10000) kindly provided by Dr. Frederic Gaymard (INRA Montpellier, France) and revealed by chimioluminescence using a secondary antibody coupled to horse raddish peroxidase (1/5000) as described (Dellagi *et al*.; 2005; Plant Journal 43(2):262-272). The membranes used for ferritin detection are the same as those used in Guiboileau *et al.* (2013). Equal loading and transfer effciency between wild type and mutant lanes was previously checked by Guiboileau *et al.* (2013). Quantification on Western blot was performed using imageJ software.

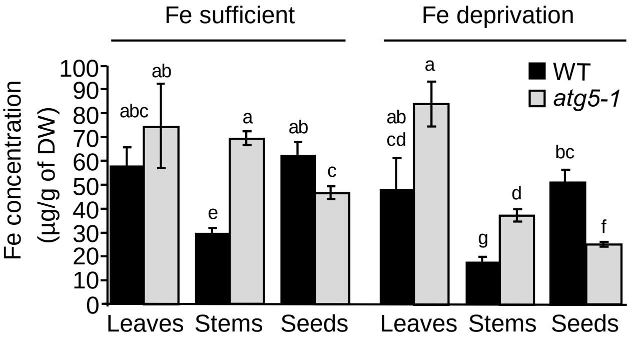

Supplemental figure S3. The increase in Fe concentration in leaves and stems of the *atg5-1* mutant is associated to a significant decrease in seed Fe concentration in *Arabidopsis* (experiment 3). Fe concentrations in leaves, stems including empty siliques, and seeds of wild-type (WT, black bars) and *atg5-1* mutant (gray bars) growing on sand/perlite (1/1) substrate watered with modified Hoagland medium. Iron was supplied (Fe sufficient) or not (Fe deprivation) during the reproductive stage. Results are shown as mean ± SE of three to four biological repeats. Different letters indicate significant differences according to a Kruskal-Wallis test ($p < 0.05$, n = 3-4) followed by a Tukey *post hoc* test.

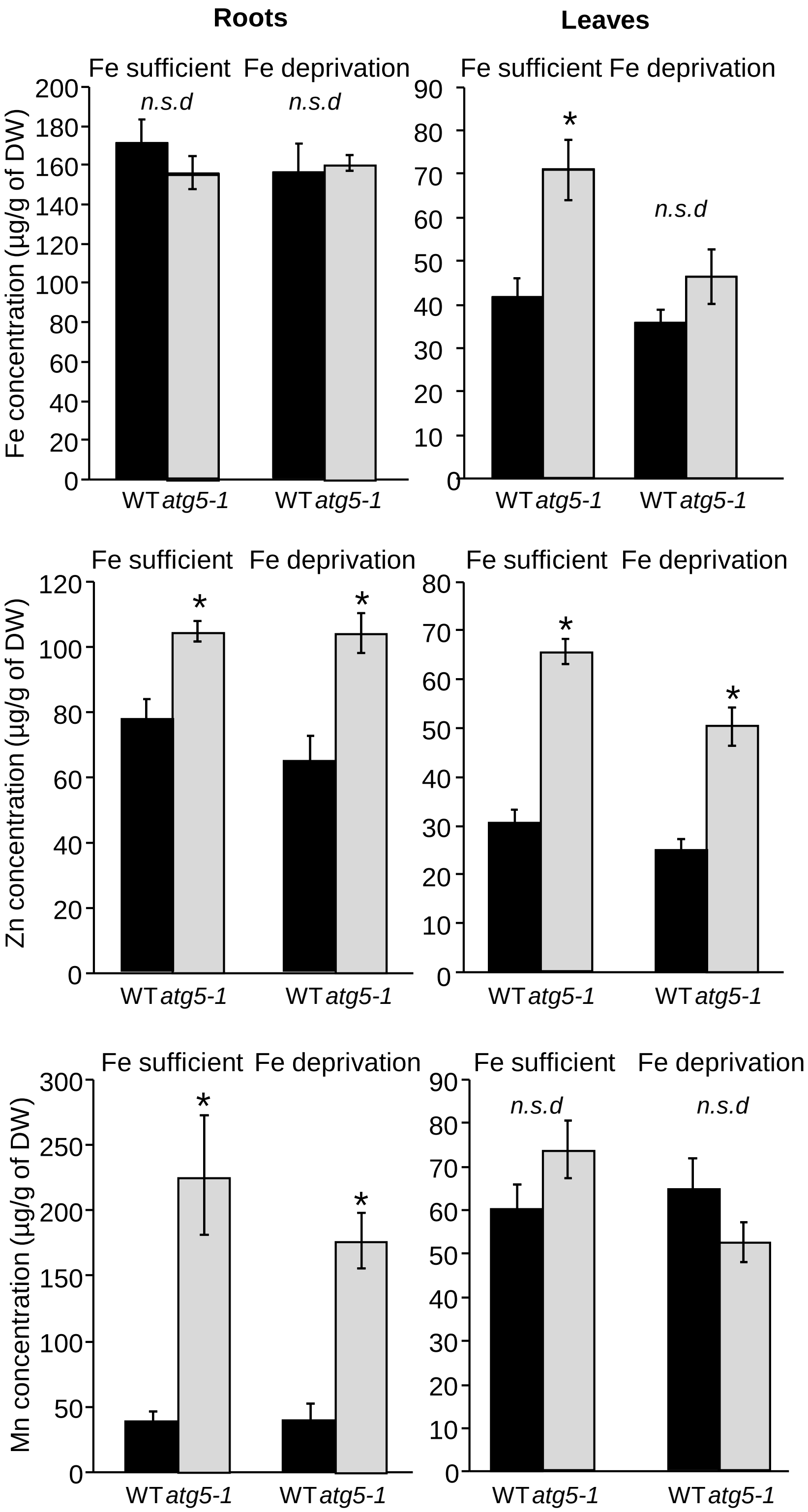

Supplemental figure S4. Fe, Zn, and Mn concentrations in roots and rosette leaves measured at the onset of seed loading in wild-type (WT, black bars) and *atg5-1* mutant (gray bars) *Arabidopsis* (experience 3). Plants were grown on sand/perlite (1/1) under Fe sufficient or Fe deprivation conditions. Results are shown as of eight biological repeats ± SE. * indicate significant differences between wild-type and *atg5-1* plants according to a Mann-Whitney test ($p < 0.05$, n = 3-4). *n.s.d* : no significant difference.

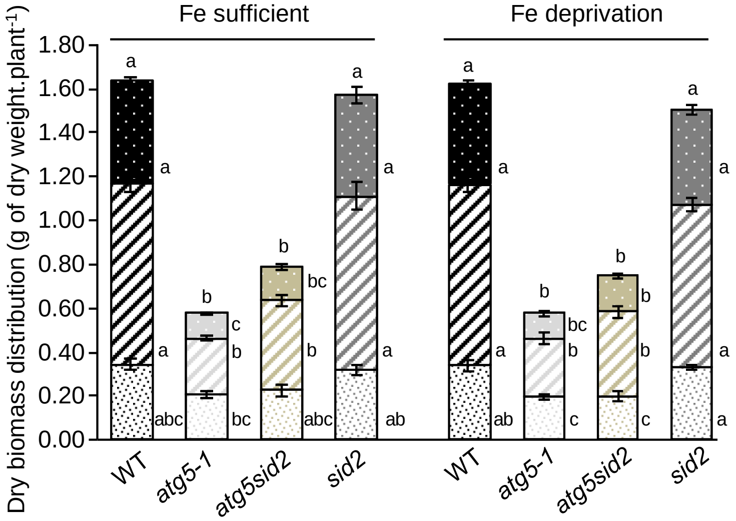

Supplemental figure S5. Total dry biomass and dry biomass distribution between organs are dramatically affected in *atg5-1* and *atg5sid2* mutants of *Arabidopsis,* even under Fe sufficient condition (experiment 2). Repartition of the dry biomass between leaves (closed squares), stems including empty siliques (diagonal stripes), and seeds (white dots) of plants growing under Fe sufficient and Fe deprivation conditions are represented for the wild-type (WT, black bars), the *atg5-1 (*light gray bars), the *atg5sid2* (brown bars), and the *sid2* (dark gray) mutant plants. Plants were grown on sand/perlite (1/1) substrate watered with modified Hoagland medium. Fe was supplied (Fe sufficient) or not (Fe deprivation) during the reproductive stage. Results are shown as mean ± SE of five to eight biological repeats. Different letters indicate significant differences between genotypes and conditions in a given organ according to a Kruskal-Wallis test ($p < 0.05$, n = 5-8) followed by a Tukey *post hoc* test.

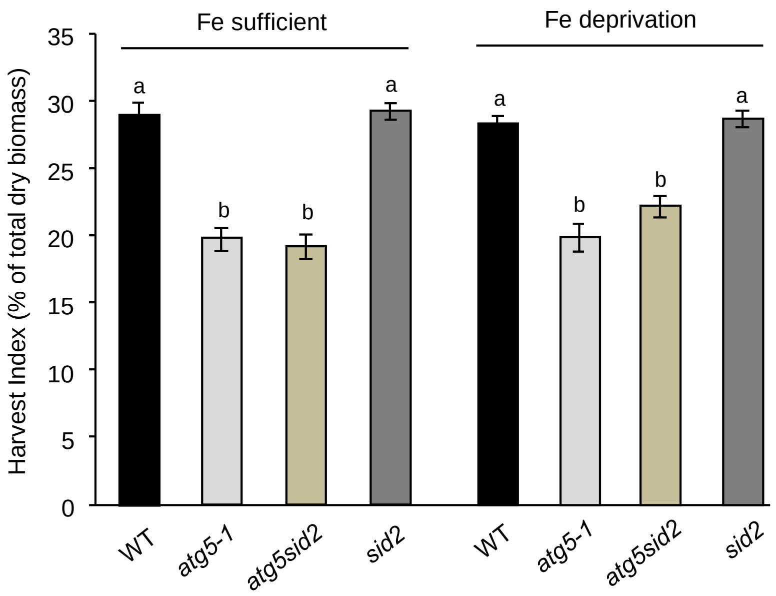

Supplemental figure S6. Harvest index is affected in autophagy deficient *Arabidopsis* even in absence of premature leaf senescence, independently of the Fe supply condition (experiment 2). The proportion of dry biomass in seeds was calculated for the wild-type (WT), the *atg5-1*, the *atg5sid2*, and the *sid2* mutant plants, as the harvest index (HI) following the equation: HI = dry weight $_{seeds}$ / dry weight $_{(leaves + stems+ seeds)}$. Plants were grown on sand/perlite (1/1) substrate watered with modified Hoagland medium. Fe was supplied (Fe sufficient) or not (Fe deprivation) during the reproductive stage. Results are shown as mean ± SE of five to eight biological repeats. Different letters indicate significant differences between genotypes and conditions according to a Kruskal-Wallis test ($p < 0.05$, n = 5-8) followed by a Tukey *post hoc* test.

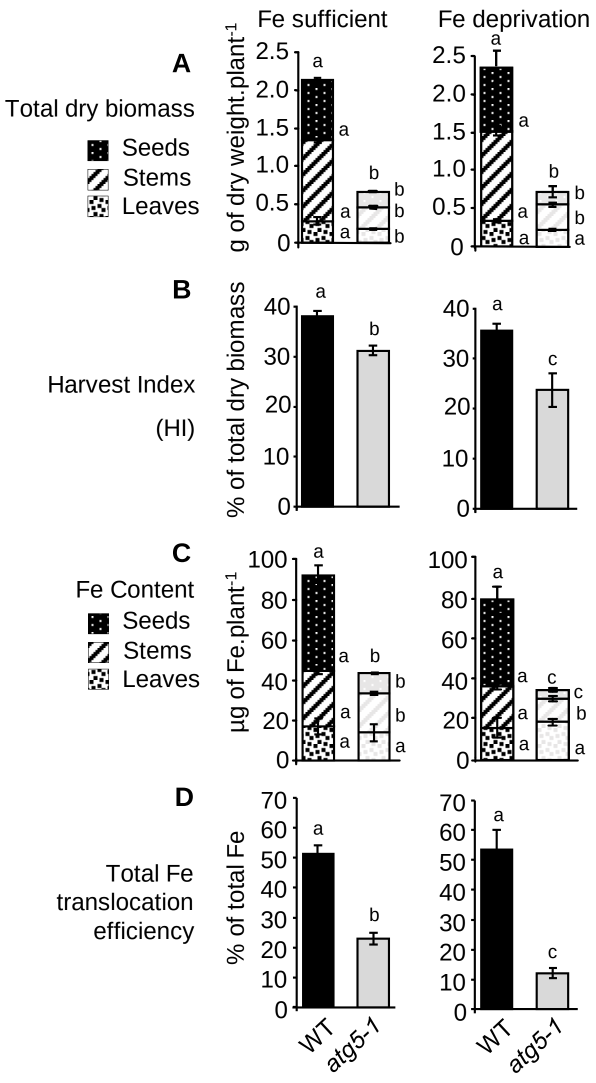

Supplemental figure S7. Total seed production and total Fe translocation efficiency are dramatically affected in *atg5-1* mutant of *Arabidopsis* even under Fe sufficient condition (experiment 3). A, Repartition of the DW between leaves (closed squares), stems including empty siliques, (diagonal stripes) and seeds (white dots) of plants growing under Fe sufficient or Fe deprivation conditions are represented for the wild-type (WT, black bars) and the autophagy mutant (*atg5-1*, gray bars). B, The proportion of dry biomass in seeds was calculated as the harvest index (HI) following the equation: HI = dry weight $_{seeds}$ / (dry weight $_{(leaves + stems + seeds)}$. C, Repartitions of the Fe content in leaves, stems including empty siliques, and seeds are represented. D, The proportion of Fe in seeds was calculated as the total Fe translocation efficiency (Fe content $_{seeds}$ / (Fe content $_{(leaves + stems + seeds)}$). Wild-type (WT) and *atg5-1* mutant plants were grown on sand/perlite (1/1) substrate watered with modified Hoagland medium. Fe was supplied (Fe sufficient) or not (Fe deprivation) during the reproductive stage. Results are shown as mean ± SE of 3 to 4 biological repeats. Different letters indicate significant differences between genotypes and conditions in a given organ according to a Kruskal-Wallis test ($p < 0.05$, n = 3-4) followed by a Tukey *post hoc* test.

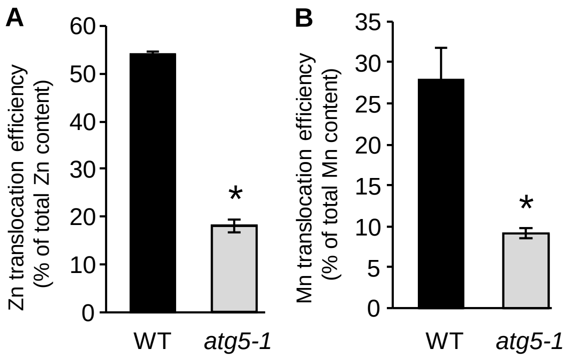

Supplemental figure S8. Translocation efficiencies of Zn (A) and Mn (B) are affected in *atg5-1* mutant of *Arabidopsis* (experiment 3). Translocation efficiencies were calculated as the ratio of micronutrient amount in seeds on the micronutrient amount in whole plant. Wild-type (WT; black bars) and *atg5-1* mutant (gray bars) plants were grown on sand/perlite (1/1) substrate watered with modified Hoagland medium containing Fe during the whole plant development, including during the reproductive stage. Results are shown as mean ± SE of three to four biological replicates. * indicate significant differences between wild-type and *atg5-1* plants according to a Mann-Whitney test ($p < 0.05$, n = 3-4).
